# Biosimilar Gene Therapy: Investigational Assessment of Secukinumab Gene Therapy

**DOI:** 10.22074/cellj.2020.6309

**Published:** 2019-07-31

**Authors:** Ali Fallah, Hajar Estiri, Elizabeth Parrish, Mansoureh Soleimani, Sirous Zeinali, Azita Zadeh-Vakili

**Affiliations:** 1 Department of Biotechnology, School of Advanced Technologies in Medicine, Shahid Beheshti University of Medical Sciences, Tehran, Iran; 2 RNAx Ltd., London, UK; 3 Cellular and Molecular Research Center, Iran University of Medical Sciences, Tehran, Iran; 4 Molecular Medicine, Pasteur Institute of Iran, Tehran, Iran; 5 Cellular and Molecular Endocrine Research Center, Research Institute for Endocrine Sciences, Shahid Beheshti University of Medical Sciences, Tehran, Iran

**Keywords:** Gene Therapy, Genetic Vectors, Monoclonal Antibody, Secukinumab, Stem Cells

## Abstract

**Objective:**

Tumor necrosis factor-alpha (TNF-α), checkpoint inhibitors, and interleukin-17 (IL-17) are critical targets in inflammation and autoimmune diseases. Monoclonal antibodies (mAbs) have a successful portfolio in the treatment of chronic diseases. With the current progress in stem cells and gene therapy technologies, there is the promise of replacing costly mAbs production in bioreactors with a more direct and cost-effective production method inside the patient’s cells. In this paper we examine the results of an investigational assessment of secukinumab gene therapy.

**Materials and Methods:**

In this experimental study, the DNA sequence of the heavy and light chains of secukinumab antibodies were cloned in a lentiviral vector. Human chorionic villous mesenchymal stem cells (CMSCs) were isolated and characterized. After lentiviral packaging and titration, part of the recombinant viruses was used for transduction of the CMSCs and the other part were applied for systemic gene therapy. The engineered stem cells and recombinant viruses were applied for *ex vivo* and *in vivo* gene therapy, respectively, in different groups of rat models. *In vitro* and *in vivo* secukinumab expression was confirmed with quantitative real-time polymerase chain reaction (qRT-PCR), western blot, and ELISA by considering the approved secukinumab as the standard reference.

**Results:**

Cell differentiation assays and flow cytometry of standard biomarkers confirmed the multipotency of the CMSCs. Western blot and qRT-PCR confirmed *in vitro* gene expression of secukinumab at both the mRNA and protein level. ELISA testing of serum from treated rat models confirmed mAb overexpression for both *in vivo* and *ex vivo* gene therapies.

**Conclusion:**

In this study, a lentiviral-mediated *ex vivo* and *in vivo* gene therapy was developed to provide a moderate dose of secukinumab in rat models. Biosimilar gene therapy is an attractive approach for the treatment of autoimmune disorders, cancers and other chronic diseases.

## Introduction

Autoimmune diseases comprise 81 clinically distinct conditions and affect approximately 2.7% of the male, and 6.4% of the female population globally. Psoriasis, celiac disease, Graves’ disease, inflammatory bowel disease, multiple sclerosis, rheumatoid arthritis, systemic lupus erythematosus, and diabetes mellitus type 1 are some common autoimmune diseases. Diagnostics and categorization of autoimmune diseases are generally difficult, and it is expected that the percentage of the people affected with autoimmune diseases will increase. Autoimmune diseases have overlapping mechanisms with the same functional cells and molecular malfunctions (1).

T helper 17 (TH17) cells are a distinct subtype of CD4+ TH cells that produce interleukin-17 (IL-17) and play a critical role in the defense against fungal and bacterial extracellular pathogens. Furthermore, TH17 cells play a core role in chronic inflammatory and autoimmune disorders, namely, multiple sclerosis, rheumatoid arthritis, psoriasis, asthma, and type 1 diabetes (2). IL-17 is a CD4+ T cell-derived cytokine that promotes inflammatory responses and is elevated in rheumatoid arthritis, asthma, multiple sclerosis, psoriasis, diabetes, and transplant rejection (3). IL-17 and IL-17Rs inhibitors have recently shown potential for universal targeting treatments to tackle autoimmune diseases (4). 

Developing monoclonal antibodies (mAbs) against IL-17 and IL-17Rs are advantageous for the biopharma industry’s fight against autoimmune diseases and cancer. Secukinumab (Cosentyx), Ixekizumab (Taltz), and Brodalumab (Siliq) are approved mAbs against IL-17 and IL-17R. A few molecules such as IL-17, checkpoint inhibitors, and tumor necrosis factor-alpha (TNF-α) are universal targets for a broad spectrum of cancers and inflammatory and autoimmune diseases. Gene therapy based biosimilars of these universal molecules will provide more available and cost-effective solutions for long-lasting diseases. Approved secukinumab is employed to treat psoriasis, ankylosing spondylitis, and psoriatic arthritis. Developing a gene therapy based biosimilar of secukinumab provides a time and cost effective, universal therapy for disease with IL-17 pathogenicity. 

In immunotherapy, neutralizing the antigen is an important step for reaching the desired therapeutic effects. A blocking antibody binds to its target molecule to directly interfere with the molecule’s function or to modify a downstream cellular effect (4). Targeting mAbs to novel antigens in the body is complex. Thus, the mAb needs an effective therapeutic dose in order to be effective. Antibodies are large, complex proteins with expensive production, purification, formulation, storage and distribution processes. Improvement and cost reduction in the process of antibody production and distribution will dramatically contribute to a reduction in the cost of immunotherapy. 

The integration of the DNA code of mAbs in cells will allow for transcription into mRNAs, and subsequently, the mRNA will produce a few thousand mAbs. This means that the patient’s cells will be working as a bioreactor, therefore manufacturing, storage, transportation, and finally administration steps will be eliminated, or at least would be reduced with the natural production of proteins inside the human body. Introducing DNA code to patient’s cells for providing intrinsic sources for the antibody is possible with gene therapy and RNA therapy (5, 6). A relatively equal number of antibodies are needed to eliminate antigens, and DNA or mRNA molecule of mAbs will eliminate a few thousand to a few million antigens with any mRNAor gene therapy. Thus, with signs of progress in gene therapy and RNA therapy platforms, in the near future, these technologies can reduce the cost of antibody-based immunotherapy. 

Gene therapy strategies are applicable in *ex vivo* and *in vivo* formats. *In vivo* gene therapy involves the systemic injection of the viral or non-viral vectors into the blood or a local injection into tissues like muscles (7). *Ex vivo* gene therapy involves cell extraction, genetic engineering of the cells and transplantation of the manipulated cells back into the body. A current approved cell-based immunotherapy, the chimeric antigen receptor (CAR) T cells, mainly rely on lentivirus-mediated *ex vivo* gene therapies (8).

With emerging stem cell technologies in clinical applications, *ex vivo* gene therapy will evolve by providing gene products releasing from manipulated stem cells. In addition to providing gene products, the engineered cells will incorporate into normal and damaged tissues and will provide additional regenerative advantages (9). Pluripotent and multipotent stem cells are an excellent carrier for *ex vivo* gene therapy. Chorionic villi mesenchymal stem cells (CMSCs) are abundant and have an immunomodulatory capability, and a high rate of division and differentiation make them unique carriers for *ex vivo* gene therapy (10). 

There are several viral and non-viral gene transfer systems for *ex vivo* and *in vivo* gene therapies. Adeno associated viruses (AAVs) are a popular format for *in vivo* gene therapy and lentiviral vectors are widely used in *ex vivo* gene therapy. Lentiviral vector features include; integration, targeting, low immunogenicity, and large transgene carrying capacity, thus they are an ideal choice for *ex vivo* gene therapy. In many *ex vivo* gene therapies like CAR T cell immunotherapy, lentiviral vectors are the main gene transfer system (11). 

Next-generation immunotherapies will play a critical role in reducing health care costs, in combination with a growing biosimilar market they will provide more cost-effective advanced therapies. A biosimilar drug is a biological medicine that is similar to a referenced and approved product and its clinical properties in terms of safety, purity and potency are the same as the reference drug. Biosimilar drugs offer less expensive treatment options for patients, therefore the shorter required time, lower-cost and high competition in biosimilar approval pathways would improve patients’ access to life-saving drugs for serious diseases such as cancer and autoimmune diseases. 

Autoimmune diseases affect the lives of patients from the emergence of the first symptoms till the end of their lives. Protein-based therapies have a 21-30-day half-life and create a huge financial burden for patients with short lasting effects. However, with RNA and gene therapy the drug can last from a few months to years and will provide a more cost-effective and painless solution (12). 

In this study, *ex vivo* and *in vivo* secukinumab biosimilar gene therapy is investigated in rat models. As with protein-based biosimilars, similarity in DNA and protein sequences is key. The aim of this study is to present a proof of concept for replacing recombinant biosimilars with gene therapy based biosimilars. Considering the function of IL-17 in the initiation and progression of many autoimmune diseases, the secukinumab antibody was selected for this research. To the best of our knowledge, there are no clinical trials for biosimilar gene therapy. Hopefully, biosimilar mRNA and gene therapy can provide more options for the biosimilar industry that will lead to lower health care expenditures.

## Materials and Methods

This study approved by The Local Ethics Committee of The Research Institute for Endocrine Sciences, Shahid Beheshti University of Medical Sciences (ir.sbmu. endocrine.rec.1395.195). 

### Dual promoter lentiviral vector construct

In this experimental study, the secukinumab protein sequences were extracted from the patent (US7807155B2) and published data by the manufacturing company (Novartis AG, Switzerland). The full DNA sequence of heavy and light chains (HC-LC) of secukinumab were synthesized as the human IgG1. antibody. The DNA sequence of HC and LC was separated by T2A self- cleavage peptide and Furin endopeptidase sequence. The secukinumab, T2A, and Furin DNA sequences were cloned after a cytomegalovirus (CMV) promoter in the single open reading frame (ORF). The vector pCDH513B1 (System Bioscience, USA) in addition to the cloned secukinumab, expressed copGFP and a puromycin resistance gene under the control of EF1 promoter as a bicistronic mRNA (13). The transfer vector (pCDH513B1) is a Tat-independent and 3^rd^ generation lentiviral vector. 

### Recombinant lentivirus production, titration, and concentration 

Inducible packaging cells (293SF-PacLV 29-6) were used for recombinant lentivirus production (14). The 293SF-PacLV cells express the CymR and rtTA2s-M2 regulators. The Rev and VSV-G genes were under the control of an inducible Tet (rtTA2S-M2) and Cumate (CymR) promoters respectively and Gag-Pol was under the control of the constitutive CMV promoter. The transfer vector was transfected after which packaging was induced with 1 µg/ml doxycycline and 30 µg/ml cumate. The transfer vector (21 µg) was transfected with a CaPo4 method with 2×10^6^ 293SF-PacLV cells in a 10 cm plate. After 14-16 hours, the transfection rate was monitored by observing GFP intensity under a fluorescence microscope (Nikon, Japan). The transfection reagents were replaced with 12-15 ml of fresh culture medium with 10% fetal bovine serum (FBS, Gibco, USA), 1 µg/ml doxycycline and 30 µg/ml cumate. At the 3^rd^, 4^th^ and 5^th^ days after transfection, the supernatant was collected and replaced with fresh medium that contained an inducer. After incubation for at least 12 hours at 4°C, precipitation at 10% polyethylene glycol (PEG, Sigma, USA) was performed followed by centrifuging (4°C, 10000 g) in order to concentrate the recombinant viruses. Titration of the recombinant viruses was done with flow cytometry on both crude and concentrated viruses (15). 

### Chorionic villi mesenchymal stem cells isolation, expansion, and characterization

After an ethical committee approval and consent from the parents, human placenta tissue was obtained under sterile conditions (at Erfan Hospital, Iran). The transfer buffer contained penicillin-streptomycin (Pen-Strep, Gibco, USA) and amphotericin B (AmphB, Gibco, USA) and was used to avoid contamination in the transfer to the lab. The fresh sample was washed 3 times with FBS supplemented with Pen-Strep-AmphB. A small amount of the chorionic tissue from below the chorionic plate was dissected out. A tiny piece of villous tissue was washed 3 times with phosphate-buffered saline (PBS, Sigma, USA) containing Pen-Strep-AmphB. After mechanical digestion with surgical scissors and scalpel, trypsin (0.5%) and collagenase type I (100 U/µL) were added to 3 ml of tissue-containing medium in a 15 mL centrifuge tube. This mixture was shaken for 30 minutes inside a 37°C incubator. The enzymes were inactivated with 500 µL of FBS, mixed thoroughly with a pipette, and centrifuged at 1200 RPM at room temperature (RT) for 5 minutes. The supernatant was carefully discarded of, the depleted cells were suspended in fresh DMEM-F12 (Gibco, USA) medium with 10% FBS and were cultured in a T75 flask. After reaching 75-80 % confluence, the first passage was done with a 1:3 ratio. The main part (80%) of the extracted cells were frozen in DMEM medium containing 20% FBS and 10% dimethyl sulfoxide (DMSO, Invitrogen, USA) and stored in liquid nitrogen. Part of the cells (20%) were characterized based on morphology, cell division, and differentiation to adipogenic and osteogenic lineages. 

### Chorionic villi mesenchymal stem cells Flow cytometry analysis

Using cell surface biomarkers cells were analyzed. To confirm cells were CMSCs the use of CD73, CD90, CD44, and CD105 were regarded as specific mesenchymal stem cell markers, and CD34, CD11b were regarded as negative markers. A small part of the undifferentiated CMSCs (passage 3, 10<sup>5</sup> cells) were checked using BD FACS Calibur flow cytometry (BD Biosciences, US) for the expression of CMSC surface markers using cell- specific antibodies. After the addition of the recommended concentration of antibodies, cells were incubated in the dark at the RT for 30-60 minutes; flow cytometry analysis was performed, and the data was analyzed using FlowJo (version 7.6.1) software.

### CMSC differentiation, cells transduction, and cell proliferation assays 

For adipogenic differentiation of CMSCs, cells were cultured in DMEM-F12 containing 10% FBS, 0.5 mM isobutylmethylxanthine (IBMX, Invitrogen, USA), dexamethasone (1 µM, Invitrogen, USA), insulin (10 µg/ mL, Sigma, USA), and indomethacin (100 µM, Sigma, USA). For osteocyte differentiation, cells were cultured in DMEM-F12 containing 10% FBS (Gibco, USA), dexamethasone (1 µM), ß-glycerol-phosphate (0.2 mM, Sigma, USA), and ascorbic acid 2-phosphate (50 µg/ml, Sigma, USA). 

CMSCs and Chinese hamster ovary (CHO) cells at 30% confluence were seeded in T75 flask in DMEM-F12 and supplied with 10% FBS. Recombinant lentiviral particles without secukinumab were used for the first group, while secukinumab expression particles were used in the second. The spinfection protocol (1500 rpm for 1.5 hours) was applied for CMSCs and CHO transduction with a multiplicity of infection (MOI) equal to 5. After 24 hours the virus-containing medium was replaced with fresh medium (DMEM-F12 with 10% FBS). After 72 hours cell viability and transduction efficiency were evaluated under an inverted light and fluorescence microscope (Nikon, Japan). For the purpose of selection, transduced cells were treated with 1.5 µg/ml puromycin, 72 hours after transduction.

The cell viability was evaluated with an MTT assay after puromycin selection in both non-transduced cells, and those transduced with an MOI of 1, 5 and 10. About 7×10^3^ cells were cultured per well, in 96 well plates. After 24 hours, MTT reagents were added and incubated for 4 hours. With the addition of DMSO, the MTT reaction was terminated. MTT was quantified by using absorbance readings via the microplate reader (BioTek, USA). 

### Quantitative polymerase chain reaction 

Total RNA was extracted from transduced and nontransduced CMSCs and CHO cells using an mRNA extraction kit (Qiagen, Germany) according to the manufacturer’s protocol. Real-time polymerase chain reaction (PCR) was carried out with 0.5 µg of extracted RNA and an SYBR Green-based master mix (*In vitro* gen, USA) in CFX96 Touch qRT-PCR machine (Bio-Rad, USA). Data was calculated as the ratio of mean threshold cycles of targeted human exogenous genes to human endogenous *GAPDH*. The specificity of the PCR product was assessed by verifying a single peak on the respective melting curve analysis. 

### Western blot and *in vitro* ELISA analysis

After transduction and selection of CMSCs and CHO cells, the supernatant from both types of cells were collected and purified using a protein A purification column. Purified, and unpurified supernatants, as well as concentrated lysates were resolved using sodium dodecyl sulfate-polyacrylamide gel electrophoresis (SDS-PAGE) and transferred to a nitrocellulose membrane. The membrane with transferred proteins was probed with a rabbit anti-human IgG antibody (Abcam, UK), washed, and incubated with a secondary HRP antibody goat anti-rabbit (Abcam, UK) conjugated with HRP for 30 minutes at RT. Subsequently, the membrane was washed 3 times with tris-buffered saline and Polysorbate 20 (TBS-T, BioRad, USA) and was incubated with enhanced chemiluminescence substrate for HRP for 1 minute. Finally, the membrane exposed to X-ray film for autoradiography. Part of the supernatant was collected from the CHO and CMSCs was transduced with recombinant viral particles to quantify *in vitro* mAbs expression. Supernatant samples were collected on days 7, 14, 21 and 30, and assayed using an ELISA kit (Abcam, UK).

### Secukinumab

#### *In vitro* functional bioassay 

The inducing effect of IL-17 on IL-6 production in human fibroblast was used in a functional bioassay of secukinumab. In this experiment, secukinumab was collected and purified from transduced CMSC cells. Cultured human dermal fibroblasts were incubated with IL-17A (15 ng/ml) in the presence of increasing concentrations of the secukinumab antibody. After 48 hours the production of IL-6 in these cells was quantified using an ELISA kit (Abcam, UK) as an indicator of secukinumab functionality.

### Intravenous delivery of transduced CMSCs and recombinant viruses

In this experimental study 50 adult female Wistar rats aged 6-8 weeks were purchased (Pasture Institute, Iran). Housing and handling of the rats were done based on standard animal laboratory protocols. Rats were divided into five groups that were injected with either PBS, recombinant GFP lentiviruses (rLV-GFP), recombinant secukinumab viruses (rLV-Secu), CMSC cells transduced with recombinant GFP lentiviruses (CMSC-rLV-GFP), or CMSC cells transducted with recombinant secukinumab lentiviruses (CMSC-rLV-Secu). 

An intravenous injection of 2×106 genetic engineered cells per rat was used for *ex vivo* gene therapy. For *in vivo* gene therapy 3×10^8^ VSV-G pseudo typed recombinant lentivirus particles were injected per rat. The cells and viruses were injected into the lateral tail veins of the female rats with an insulin syringe. Both gene therapy results were checked for two months and we collected blood from the rats on days 7, 15, 30, 45 and 60. Up to 1.5 ml of blood was collected from the Rats at each time point for serum separation. Rat serum was tested by quantitative ELISA using the anti-human IgG1 antibody kit (Abcam, UK).

### Statistical analysis

Our research is an interventional study in a rat model. The data is expressed as mean values ± SD. Student’s t test was performed for survival data. P=0.05 were to be used as the threshold for statistical significance in these study. The statistical analysis was carried out using SPSS version 25 (IBM SPSS Statistics for Windows, version 25.0. Armonk, NY: IBM Corp.). 

## Results

### Construction of lentiviral-based bicistronic antibody expression vector 

**Fig.1:**
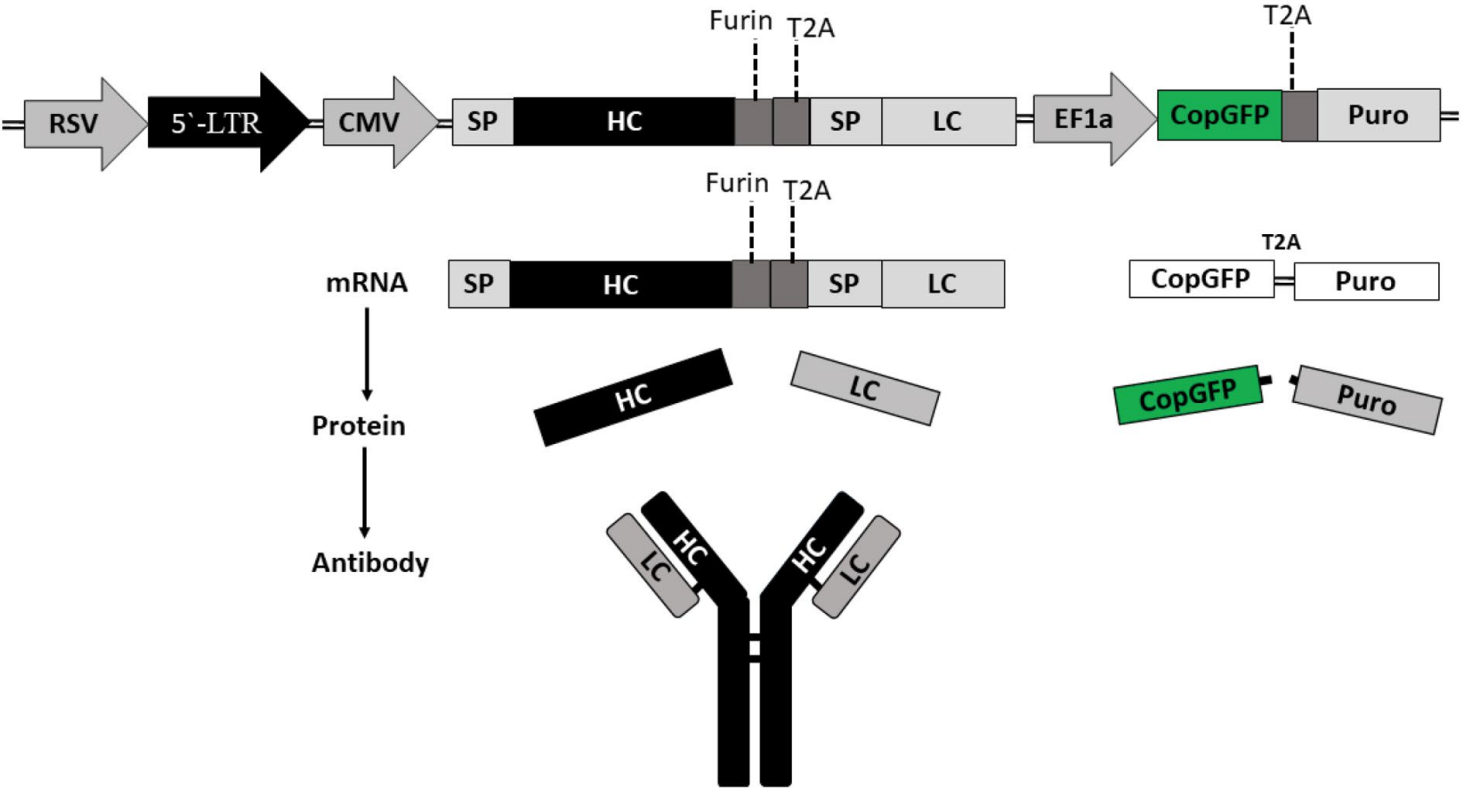
Transfer vector features and secukinumab transcription, translation and assembling. Schematic illustration of the third-generation lentiviral transfer vector with dual-promoter. This vector, after transduction, will express GFP and a puromycin selection marker. The recombinant lentiviral vector will produce two separate bicistronic mRNAs. This will lead to four proteins being produced after transduction. The secukinumab HC and LC proteins assemble as a human IgG1-Kapa antibody. RSV; Respiratory syncytial virus, CMV; Cytomegalovirus, SP; Signal peptide, LC; Light chain, and HC; Heavy chain.

Secukinumab heavy and light chain DNA sequences were synthesized with a Furin proteolytic cleavage site, a GSG- linker motif, and a T2A self-cleavage peptide between two chains. The resulting single ORF was cloned using the pCDH513B lentiviral vector. Gene cloning was confirmed through sequencing and restriction enzyme digestion. The transfer vector had dual promoters to express secukinumab under the CMV promoter and GFP and puromycin under the human EF1a promoter. The lentiviral vector produces two mRNAs and four separate proteins total, after transduction. The footprint of T2A will be removed by means of the signal peptide and furine peptidase activity. Figure 1 shows a schematic illustration of the transfer vector, as well as the transcription, translation and mAb assembly process.

### Recombinant lentiviral production, concentration, and titration:

**Fig.2:**
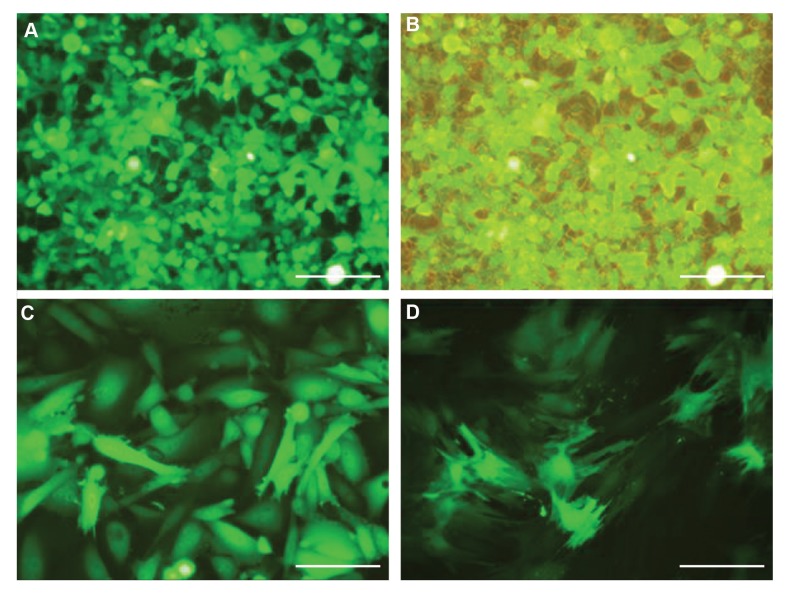
Cells transfection and transduction. **A, B.** The transfection efficiency of LV-SF9 packaging cell line with CaPo4 protocol. Imaging of GFP-specific fluorescence with about 90- 95% transfection rate, **C.** Imaging of transduced CHO, and **D.** Imaging of transduced CMSC cells with secukinumab transfer vector that were selected with puromycin. Use of GFP helped with visualization during all transfection and transduction steps and puromycin was used for selection of transduced cells (scale bar: ×100). CHO; Chinese hamster ovary and CMSC; Human chorionic derived mesenchymal stem cells.

LV-SF9 cells were transfected with CaPO_4_ resulting in 90-95% GFP positive cells (Fig .2A, B). Based on FACS titration, more than 5-6×10^6^ recombinant particles were found in the crude supernatant. After virus concentration with PEG precipitation, the total titration reached to 2-3×10^8 ^particles/ml. LV-SF9 is a suspension culture adapted cell line that is developed for large scale lentiviral packaging. The Helper gene and envelope gene products will be made following the addition of the inducer to the culture medium and only the transfection of the transfer lentiviral vector is needed. 

### Stem cells isolation, characterization, and transduction 

**Fig.3:**
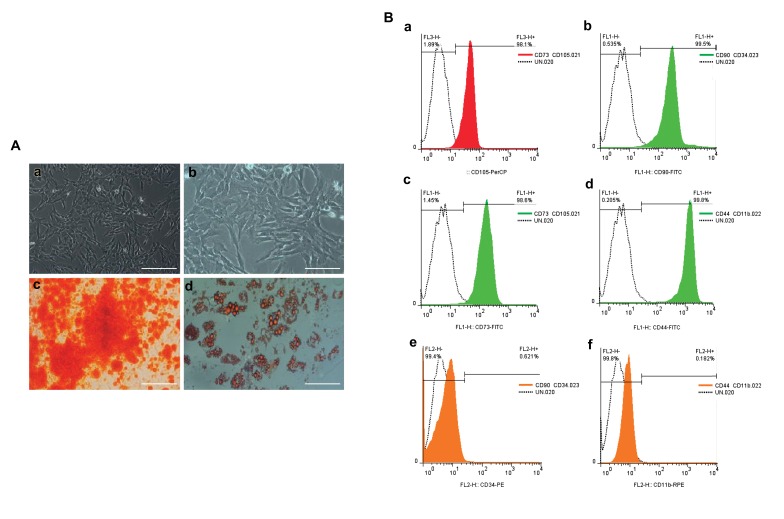
Human chorionic derived mesenchymal stem cells (CMSCs) cell morphology, differentiation and flow cytometry. **A.** Inverted microscope image of CMSCs with ×100 magnification (a), and a ×200 magnification (b). The mesenchymal morphology is clear in both figures, the CMSCs osteogenic (Alizarin Red) and adipogenic (Oil Red) fates (c, d) and *B.* Flow cytometry analysis results for CMSCs positive markers [CD44 (a), CD73 (b), CD90 (c), CD105 (d)], and negative markers [CD34 (e) and CD11b (f)].

A MOI of 5 was applied for the transduction of CHO and CMSC cells, and about 65-70% transduction rate was confirmed by observation of GFP under a fluorescent microscope after 72 hours. Completely purified transducted CHO and CMSC cells was achieved by treating the cells with puromycin for selective culture (Fig .2C, D). The application of puromycin is important for preclinical uses as it leads to high purity of manipulated cells and for optimizing the dosage for gene therapy. GFP helped with visualization in every step from transfection, and transduction, to selection. Use of GFP and puromycin are not allowed for clinical applications but help for optimization in pre-clinical studies. CMSCs were isolated from fresh placenta chorionic villi tissue. After primary cell confluency of 80%, a large part of the cells (90%) were stored and a small part of the cells (10%) were treated for characterization. Isolated cells were confirmed based on morphology (Fig .3Aa, b). Osteogenesis and adipogenesis through the differentiation of these cells were confirmed (Fig .3Ac, d). It is important that stem cells function as more than the carrier of genes and integrate into the host tissues. CMSCs in *ex vivo* gene therapy will be able to differentiation based on extrinsic signals that will be received after homing. Flow Cytometry analysis (Fig .3B) showed a high rate of cells positive for CD44, CD73, CD90, and CD105 specific markers and a low rate of cells with negative markers (CD34, CD11b). The results indicated the high purity of isolated CMSCs and demonstrated the efficacy of this protocol. 

### *In vitro* gene overexpression assessment at the mRNA level 

**Fig.4:**
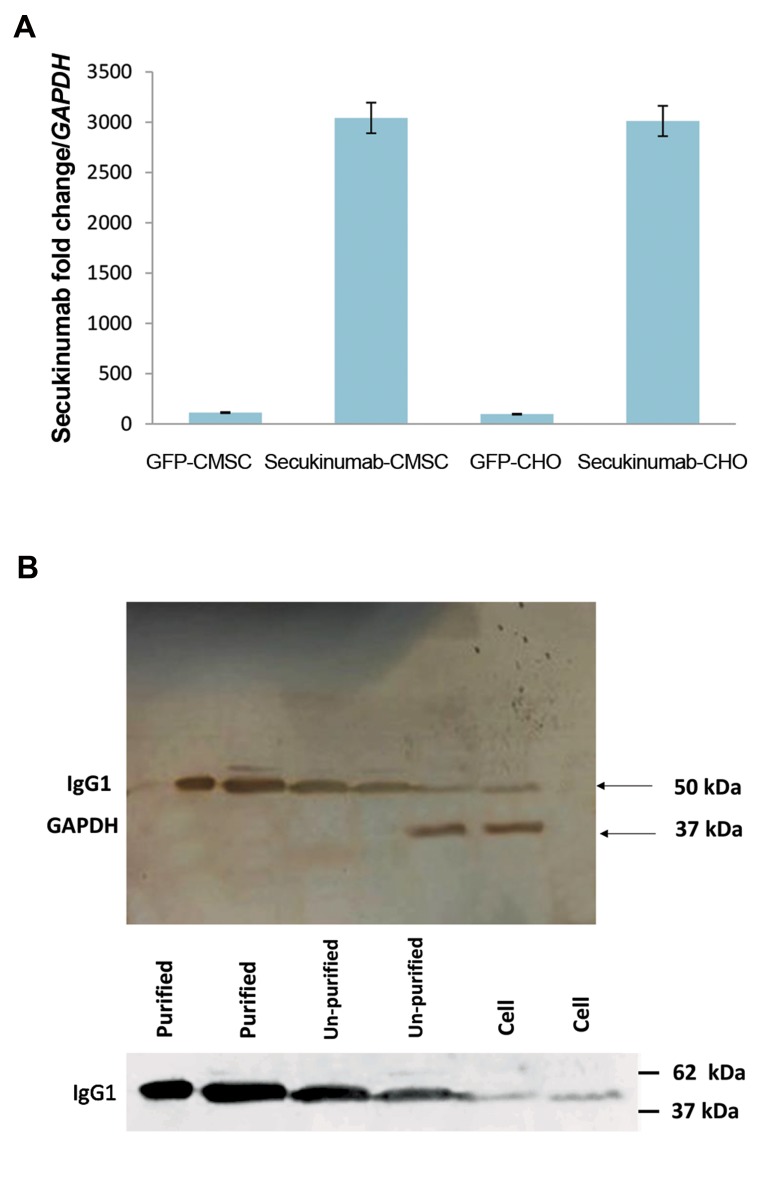
qRT-PCR and western blot. **A.** Comparison of secukinumab gene expression by qRT-PCR in CHO cells and CMSCs. Cells transduced with secukinumab transfer vector or GFP recombinant lentiviruses and **B.** WB test results from both CMSCs and CHO cells. Purified secukinumab with protein A from the supernatant of CMSCs (line 1) and CHO cells (line 2). Unpurified but concentrated supernatant secukinumab from CHO cells (line 3) and CMSCs (line 4). Concentrated lysate total protein from CHO cells (line 5) and CMSCs (line 6) for comparison with the control GAPDH protein. WB was done with the primary antibody against the FC domain of the HC of human IGg1. qRT-PCR; Quantitative real-time polymerase chain reaction, CHO; Chinese hamster ovary, WB; Western blot, CMSCs; Human chorionic derived mesenchymal stem cells, GFP; Green fluorescent protein, and HC; Heavy chain.

To assess the amount of mAb gene expression, mRNA levels were measured using quantitative real-time polymerase chain reaction (qRT-PCR) for both CHO and CMSC cells. The qRT-PCR results confirmed the expression of secukinumab in CHO and CMSC cells but not in GFP- only transduced control cells. Based on results shown in (Fig .4A) transcription of secukinumab mRNA was dramatically increased in both CMSCs and CHO cells.

### *In vitro* mAb expression, cell viability assay, and IL17 bioassay 

**Fig.5:**
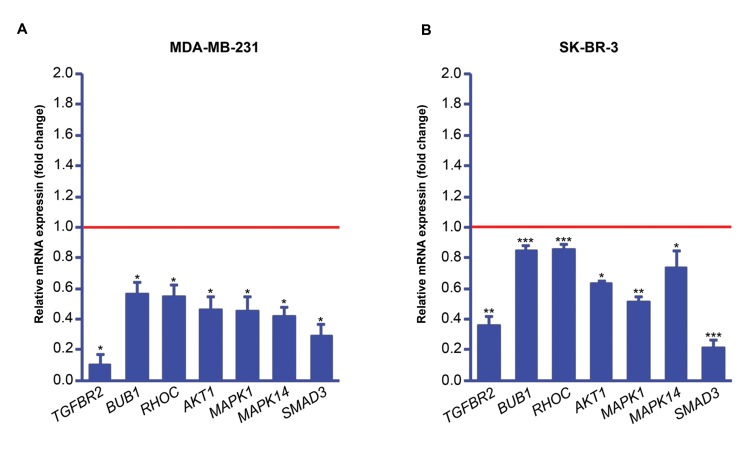
Cell viability MTT assay and secukinumab bioassay. **A.** MTT assay results for CMSCs before and after transduction with secukinumab transfer vector. As shown in the figure, transduction with an MOI of 1,5, or10 has no significant effect on CMSC viability and **B.** ELISA test results of IL-6 on human primary dermal fibroblast in increasing concentrations of secukinumab in the presence of recombinant human Interleukin-17A (15 ng/ml). After 72 hours reduced IL-6 secretions from fibroblasts confirmed the inhibitory activity of secukinumab on human IL-17. CMSCs; Human chorionic derived mesenchymal stem cells, MOI; Multiplicity of infection, and OD; Optical density.

*In vitro secukinumab* expression at the protein level was confirmed with WB (Fig .4B). After the transduction of CHO cells and CMSCs, the viability of the CMSCs was checked using MTT assay (Fig .5A). These results clearly showed that transduction and mAb production didn’t affect the physiological viability of CHO cells and CMSCs.

Based on the fact that IL-17 induces IL-6 expression in human dermal fibroblasts, the ability of secukinumab to neutralize human IL-17A and inhibit IL-17A-induced IL-6 production was assessed. For the IL-17 bioassay, we applied ELISA as shown in Figure 5B. This result confirms that our secukinumab is fully functional and can bind successfully to IL-17A to inhibit this ligand from attaching to IL-17R. 

**Fig.6:**
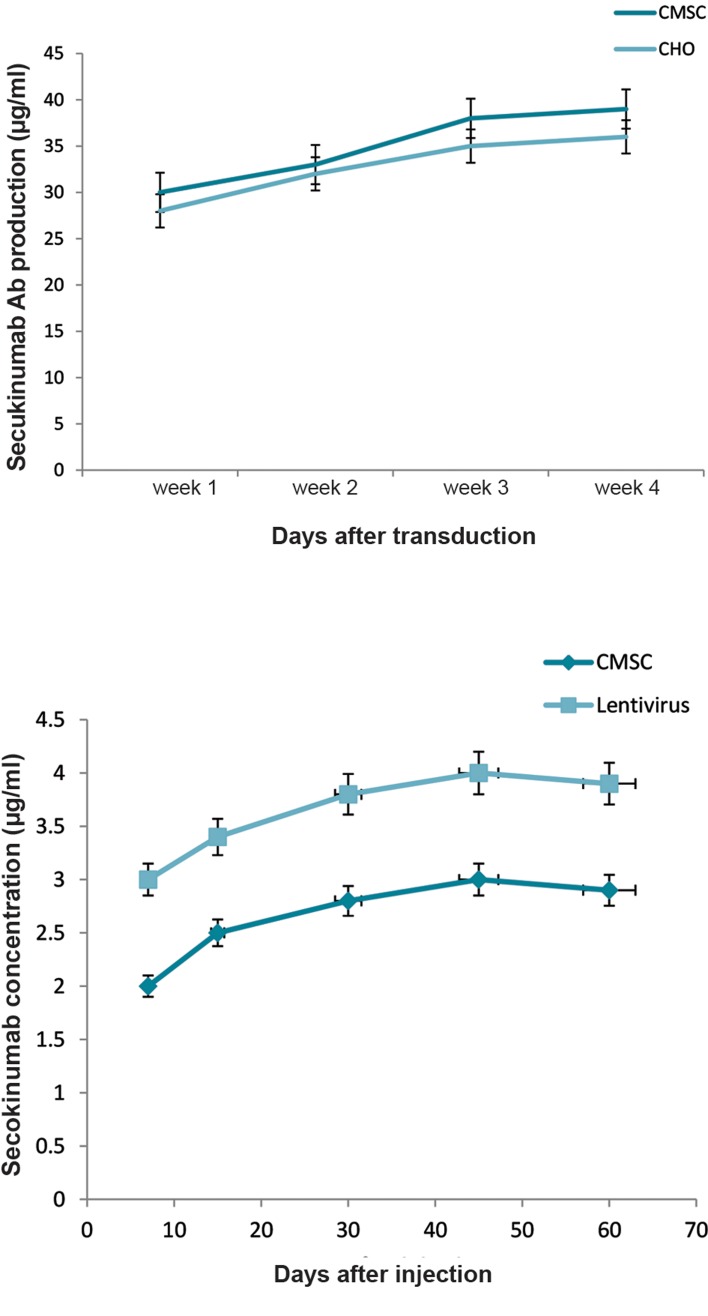
Secukinumab *in vitro* and *in vivo* ELISA. **A.** *In vitro* ELISA tests of secukinumab production from transducted CHO cells and CMSCs with the secukinumab transfer vector. Stem cells showed slightly higher mAbs production in comparison with CHO cells. Sampling was done 4 times a month and **B.** Rat serum ELISA results with five blood samples taken after treatment during the two-month duration. Secukinumab concentration resulting from *in vivo* lentivirus (orange) gene therapy is higher than CMSC-mediated *ex vivo* (blue) gene therapy. ELISA; Enzyme-linked immunosorbent assay, CHO; Chinese hamster ovary, and CMSCs; Human chorionic derived mesenchymal stem cells.

The supernatant of transduced cells was collected 7 days after transduction and puromycin selection. These cells were used as the source of the secukinumab antibody protein expression tests. These results demonstrated that secukinumab mRNA translation, assembly, and secretion as kappa-IGg1 is correct and detectable by anti-human Igg1 Fc antibody in both solutions through ELISA (Fig .6A) and fixed on a membrane using WB (Fig .4B). 

### Systemic gene therapy

Systemic administration of recombinant viruses and genetically engineered CMSCs in rats lead to secukinumab overexpression and its release in their bloodstream. Our evaluations confirmed that *ex vivo* gene therapy provided 2-3 µg secukinumab per ml of rat blood serum and *in vivo* gene therapy was shown to provide 3-4 µg/ml of serum (Fig .6B). Secukinumab epitope mapping revealed that this mAb cannot bind to mouse and rat IL-17. This is a big challenge in preclinical studies of secukinumab and other biosimilars. A few humanized animal models like TNF-αlpha (GenOway, France) are available for the preclinical study of biosimilars. In the case of secukinumab, the model allows for an *in vivo* efficacy and safety assessment of anti-human IL-17A. With regards to this point, we only checked the bioavailability of secukinumab in the rat model in this study. 

## Discussion

In the present study we developed a novel therapeutic strategy involving the expression of a biosimilar antibody, secukinumab, through a lentivirus-based, stem cell therapy, and vector-mediated gene therapy in a rat model. We showed that lentivirus-mediated secukinumab expression is possible with relative therapeutic effects comparable to protein therapy both *in vitro* and *in vivo*. 

Lentivirus vectors, with highly efficient *ex vivo* and *in vivo* transduction, provide excellent gene transfer systems. The enveloped lentiviruses used in this research allowed us to target any cell receptor with a natural or synthetic ligand. Incorporating the GFP reporter gene allowed for monitoring of all the steps of cell engineering; puromycin provided absolute purity in the resulting engineered cells. For clinical applications, incorporating recombination systems like Cre-LoxP could allow us to remove both the fluorescent and puromycin DNA sequences after cell manipulation and before clinical administration. Inducible packaging cells with serum-free and sustainable cell culture conditions create a closer product to commercial gene therapy products (14). 

CMSCs have a high *in vitro* differentiation potency and a high level of stem cell marker expression, as such they are applicable as the base for *ex vivo* gene therapy. The DNA sequence of mAbs was integrated into the genome of CMSCs, therefore homing and differentiation of these cells directly into the body can provide a long- lasting source of therapeutic proteins. CMSCs with their immunomodulatory properties and high proliferation rates are promising cellular resources for regenerative medicine. Based on our results, there is no significant difference between mAb production by CHO cells, the predominant host used to produce therapeutic proteins and CMSCs. A comparison of *in vitro* expression between CMSCs and CHO cells showed that CMSCs can produce a comparable amount, 30-40 µg/ml of secukinumab, in established cell lines using the same vectors and sequences.

When comparing ELISA tests of *ex vivo* and *in vivo* secukinumab gene therapy, CMSCs provide more stable expression at 2-3 µg/ml of secukinumab. In comparison, direct lentivirus injection and *in vivo* gene therapy provided 3-4 µg/ml of secukinumab but with more variation over time. *In vitro* and *in vivo* mAb expression assays showed that we could apply gene therapy for expression of sustainable recombinant proteins and mAb in the patient’s body. Stem cells as a source of mAb production, with their tumor-tropic properties and unique ability to cross the blood-brain barrier (BBB), will be an alternative carrier for cancer and especially brain cancer treatment. 

*In vivo* antibody gene therapy was first attempted by means of Adenoviruses. Several research papers showed stable *in vivo* expression of mAbs with a wide range of long-term concentrations ranging from 50 ng/ml to 40 µg/ml (16, 17). Another successful vector for mAb gene therapy is Adeno-associated virus (AAV) with a range of 10-400 µg/ml even 6 months after administration (18, 19). Non-viral vectors like naked DNA, plasmid, minicircle, and mRNA delivery are the alternative approaches and produce about 1-300 µg/ml mAbs based on delivery dosage, the frequency of administration and the nature of the nucleic acid (20). 

With the current approval of lentivirus and CAR T cell products in the USA and the anticipated results in ongoing clinical trials, CAR T has emerged as a powerful viral gene therapy vector (11). Several mAb gene therapies with lentiviral vectors provide long-lasting mAbs titers in blood serum with a range of 1-3 µg/ml for more than 7 months. When comparing these preexisting study data with our results, a single dose of our gene therapy provided a relatively high level of secukinumab in the rat serum (21, 22). AAVs are impressive gene therapy vectors, however, lentiviral vectors have more stable and steady expression and additionally provide a more reliable system for therapeutic use. 

*Ex vivo* mAb gene therapy was successful in fibroblast *ex vivo* gene therapy, providing 1-2 µg/ml mAbs in the blood serum (23). The next experiment, with mesenchymal and neural stem cells, provided alternative approaches for *ex vivo* mAbs gene therapy allowing for about 1-5 µg/ml mAb in the serum (24, 25).

Like other mesenchymal stem cells, CMSCs with their immunomodulatory and cancer cell tropism provided a more efficient platform for *ex vivo mAb gene therapy. The differentiation potential of this type of stem c*ell allows for integration and adoption of these cells in the cancer environment and long-term mAb expression that is critical for some cancers like breast cancer and gliomas. Approved secukinumab serum concentrations were 44.5 µg/mL for Cosentyx 300 mg and 22.2 µg/mL for Cosentyx 150 mg. In this case, a single administration of secukinumab via *ex vivo* and *in vivo* gene therapy resulted in a 3-4 µg/mL titration, that revealed these gene therapies need improved serum concentrations for human application (26). In comparison with the current recombinant protein therapy, gene therapy is a more durable and sustainable source of secukinumab treatment. Biosimilar secukinumab gene therapy resulted in significant and prolonged antibody expression with only a single dose. Based on the definition of a biosimilar i.e. a biological medicine that is an almost identical copy of an existing authorized biological medicine, we expected that secukinumab’s biosimilar gene therapy would have the same clinical efficacy in comparison with the approved recombinant version.

## Conclusion

The high cost in the development of advanced therapies for patients can be countered by novel approaches such as, biosimilars gene therapy and mRNA biosimilars therapeutics. These technologies can provide a cost- effective and reliable approach for both the public and private healthcare systems. Engineered CMSCs and recombinant viruses can be a source of sustained expression of mAbs *in vivo*. This study showed that both *in vivo* and *ex vivo* gene therapy are effective platforms for the production of therapeutic mAbs. The approval of *in vivo* gene therapies e.g. Glybera (alipogene tiparvovec), and *ex vivo* gene therapy e.g. Kymriah (tisagenlecleucel), allow for incorporation of novel gene therapies and play a vital role in the future of the healthcare systems.
